# Prevalence of goiter among children in Ethiopia and associated factors: a systematic review and meta-analysis

**DOI:** 10.1186/s12889-019-7505-7

**Published:** 2019-08-29

**Authors:** Getenet Dessie, Desalegne Amare, Amare Belachew Dagnew, Henok Mulugeta, Dessalegn Haile Kassa, Ayenew Negesse, Getachew Mullu Kassa, Fasil Wagnew, Sheikh Mohammed Shariful Islam, Sahai Burrowes

**Affiliations:** 1 Department of Nursing, School of Health Science, College of Medicine and Health Science, Bahir Dar University, P.O. Box 79, Bahir Dar, Ethiopia; 2 Department of Nursing, College of Health Science, Debre Markos University, P.O. Box 269, Debre Markos, Ethiopia; 3 Department of Human Nutrition and Food Science, College of Health Science, Debre Markos University, P.O. Box 269, Debre Markos, Ethiopia; 4 College of Health Sciences, Debre Markos University, P.O. Box 269, Debre Markos, Ethiopia; 5 Institute for Physical Activity and Nutrition (IPAN), Deakin University, Geelong, Australia; 6 Burwood, Australia; 7 Public Health Program, College of Education and Health Sciences, Touro University California, 1310 Club Drive, Mare Island, Vallejo, CA 94592 USA

**Keywords:** Children, Ethiopia, Goiter, Meta-analysis

## Abstract

**Background:**

The distribution of goiter among children and its risk factors are not well investigated in Ethiopia. Therefore, this systematic review and meta-analysis was designed to determine the pooled prevalence of goiter and its associated factors among children in Ethiopia.

**Methods:**

Electronic web-based searches of PubMed, Google Scholar, EMBASE, and the World Health Organization’s Hinari portal (which includes the SCOPUS, African Index Medicus, and African Journals Online databases) were conducted to find primary studies. Relevant data were extracted and descriptive summaries of the studies were presented in tables. The I^2^ statistic was used to assess heterogeneity across studies. Funnel plot asymmetry and Egger’s tests were used to check for publication bias. A random effects model was used to estimate the pooled prevalence of goiter. Odds ratios (OR) with 95% Confidence Intervals (CI) were also used to determine the association of identified variables with goiter. All statistical analyses were conducted using Stata version 14 software.

**Results:**

Our search identified 982 studies, of which, 19 articles were eligible for inclusion in the final meta-analysis. The pooled estimate of goiter among children in Ethiopia was 40.50% (95% CI: 33.6–47.40). The regional distribution of goiter ranged from 44.22 (95% CI: 17.44–71) in Southern Nations Nationalities and Peoples’ Region, to 32.79% (95% CI: 19.86–45.73) in Benishangul Gumez region. The prevalence of goiter among female children (44.34%) was higher than among male (32.88%) children. Goiter prevalence was also significantly higher among children who consumed vegetables three or more times per week OR = 1.3 (95% CI: 1.02–1.66); those who had family history of goiter, OR = 2.38 (95% CI: 1.9–2.99); and those whose family stored salt near to fires, OR = 1.4 l (95% CI: 1.1–1.79).

**Conclusion:**

The prevalence of goiter among children in Ethiopia was high, and endemic according to the WHO criteria. Our findings suggest the need for interventions to improve salt iodization, and for improved health education on appropriate salt storage. In addition, more research may be needed to improve our understanding of foods that increase the risk of goiter among children.

## Background

Iodine, a trace element found in the soil, is essential for synthesizing thyroid hormones that ensure the proper functioning of the thyroid and assist with cell replication [1]. Insufficient levels of iodine in the body lead not only to goiter, an enlargement of the thyroid gland, but also to a range of severe physical and cognitive impairments such as mental retardation; cretinism; miscarriage and stillbirth among pregnant women; and growth and developmental abnormalities in infants and children [2]. According to the World Health Organization (WHO), iodine deficiency is the single greatest preventable causes of mental retardation globally [3]. Children and adolescents are particularly vulnerable to iodine deficiency disorders (IDDs) [4] because of puberty-related changes in thyroid function that may increase the need for iodine [5, 6].

Diets low in iodine are the main cause of IDDs. Iodine is found naturally in soil and water but in many parts of the world, due to a history of heavy rains or glacial melt, soils have been leeched of the mineral [2, 7]. The primary strategy for reducing the prevalence of goiter and other IDDs has been to increase iodine consumption by iodizing salt [8]. National salt iodization programs are considered a simple, cost-effective means of preventing IDDs, as salt iodization is relatively easy to implement, and salt is steadily consumed by populations year round; and because countries usually have only a few large salt manufacturers who need to be incorporated into national programs [7, 9]. Such universal iodization programs have been extremely successful and are considered a global health success story and a model of public/private partnerships for promoting health [2]. However, iodine deficiency can reappear if salt iodization is interrupted as a result of demobilization of the public health authorities or the lack of interest of the salt industry [10].

The prevalence of goiter in sub-Saharan Africa has fallen over the past four decades as populations covered by salt iodization programs increased. However, despite this success, goiter and other IDDs are still a significant public health problem among children in Eastern and Southern Africa [2]. In Ethiopia, as of 2015, the national prevalence of goiter among children aged 6 to 12 was 39.9% (more than 4 million children). According to the World Health Organization/International Council for Control of Iodine Deficiency Disorders/ United Nations Children’s Fund (WHO/ ICCIDD/UNICEF) classification, both goiter prevalence and urinary iodine levels in Ethiopia indicate that the entire country is affected by iodine deficiency [11] .

One reason for the persistence of IDDs in Ethiopia is the mixed performance of the country’s salt iodization program. Since its launch in the 1980s it has achieved remarkable improvements in iodized salt coverage despite civil conflicts and economic disruptions, which compromised the quality of the program. However, there have been significant challenges in assuring that the level of iodization of salt is sufficient. Studies have found that only a small percentage of households have access to adequately iodized salt in Ethiopia. For example, a study conducted in 2017 reported that although over 89.2% of salt in Ethiopia contains iodine, coverage of adequately iodized salt remains very low (26%) [12]. This virtual absence of adequately iodized salt has contributed to the endemicity of goiter in Ethiopia.

In addition to the coverage and adequacy of salt iodization, age, socioeconomic status, and a family history of goiter have been shown to be major risk factors for goiter in low-income countries like Ethiopia [13]. Globally, women have been found to have higher rates of goiter than men [14]. Some scholars have also suggested that very high levels of consumption of cassava and cruciferous vegetables such as cabbage and kale may be associated with hypothyroidism and thyroid cancer, particularly in areas that are iodine deficient [15, 16]. Poor salt storage may also contribute significantly to low iodine levels in salt. Recent studies in Ethiopia have found that consuming loose, rather than packaged salt, and storing salt close to open fires, decrease the level of elemental iodine [17–19] in the salt and that these behaviors are common in Ethiopia.

In sum, although there has been significant progress in the reduction of goiter and related IDDs globally, they remain significant burden in Ethiopia due, in part, to poor salt storage practices and a weak universal salt iodization program. There is little consistent data about the prevalence of goiter in Ethiopia, particularly among children; and most publicly available scientific literature and reports on the topic were published before 2016 [20]. Understanding the extent of goiter and its associated factors is important for designing strategies that can reduce the burden of goiter among children in Ethiopia. This systematic review and meta-analysis, therefore, aims to synthesize recent evidence on the prevalence of goiter among children and its associated factors in Ethiopia.

## Methods

### Search strategy and appraisal of studies

This study was conducted according to the Preferred Reporting Items of Systematic Reviews and Meta-Analysis Protocols (PRISMA-P) checklist guidelines [21].

The electronic databases searched were PubMed, Google Scholar, Embase, and Hinari. Hinari is the World Health Organization (WHO) database portal for low- and middle-income countries and includes Web of Science, SCOPUS, African Index Medicus (AIM), Cumulative Index to Nursing and Allied Health Literature (CINAHL), WHO’s Institutional Repository for Information Sharing (IRIS), and African Journals Online databases. In addition, articles were also searched through a review of the grey literature available on local university shelves, and institutional repositories, and by reviewing the reference lists of already identified articles.

The key terms used for the database searches were: “epidemiology” OR “prevalence” AND “goiter” AND associated factors OR “determinant factors” AND “child” OR “children” AND “Ethiopia”. These search terms were pre-defined to allow a comprehensive search strategy that included all fields within records and Medical Subject Headings (MeSH terms), in order to expand the search in an advanced PubMed search (Appendix). Following the search protocol described by Negesse et al. [22], we also used Boolean operator in our search as they outline in their paper: “within each axis we combined keywords with the ‘OR’ operator and we then linked the search strategies for the two axes with the ‘AND’ operator to search studies conducted specifically for each region in the country”. Electronic database searches were conducted from September 1, 2018 to November 10, 2018.

### Inclusion and exclusion criteria

All English-language, full-text articles for studies conducted in Ethiopia, published from 2000 to November 10, 2018, in peer-reviewed journals or found in the grey literature were eligible for inclusion. Goiter was defined according to the internationally accepted definition: grade 0, no goiter; grade 1, thyroid palpable but not visible; grade 2, thyroid visible with neck in normal position [23]. All studies that reported the prevalence of goiter in children were eligible for inclusion. Studies that did not report specific outcomes for goiter quantitatively were excluded.

### Data abstraction procedure

Titles and abstracts were screened based on the predefined criteria. Retrieved articles were evaluated based on their title, objectives, and methodology. At this stage, irrelevant articles were excluded and the full text of the remainder articles was reviewed for inclusion. Articles that fulfilled the prior criteria were used as source of data for the final analysis.

### Quality assessment of individual studies

The database search results were exported and duplicate articles were removed using EndNote software (version X7; Thomson Reuters, New York, NY). The Newcastle-Ottawa Scale (NOS) criteria was used for quality assessment of selected studies before analysis [24]. Two independent reviewers critically appraised each individual article using the NOS. Discrepancies between reviewers were resolved by discussion and by including a third reviewer. The average of two independent reviewer’s quality scores was used to determine whether the articles should be included. Articles with methodological flaws or incomplete reporting of results in the full text were excluded from the final analysis.

### Outcome measurements

The main aim of this review was to determine the pooled prevalence of goiter among children in Ethiopia. The prevalence was measured as the number of children with goiter in the study divided by the total number of children in a study multiplied by 100. For analysis of the secondary outcomes (factors associated with goiter), we extracted data on factors that had been found to be related to goiter in the literature, such as the consumption of vegetables, a family history of goiter, consumption of packaged vs. loose salt, and salt stored near to a fire. The other criteria for selecting variables was how frequently they were reported in studies included in the meta-analysis. In examining factors associated with goiter, data were extracted from the primary studies in the form of two-by-two tables and a crude odd ratio (OR) was calculated to determine the association between each of the independent variables and having a goiter.

### Data analysis

Information on the studies’ characteristics such as publication year, study geographic region, study design, sample size, diagnostic criteria, percentage of goiter in children, and the age-range of children were extracted from each study using a Microsoft Excel spreadsheet template. Then, data were transferred to Stata (version 14; Stata Corp, College Station, TX) for further analysis. Heterogeneity across studies was checked using the inverse variance (I^2^) and Cochran Q statistics and the cutoffs of 25, 50, and 75% were used to declare the heterogeneity as low, moderate, and severe respectively [25]. Because the test statistic indicated significant heterogeneity among studies (I^2^ > 70%, *p* < 0.05), a random effects model was used to estimate the pooled prevalence of goiter with 95% confidence interval (CI).

In addition, sub-group analysis by region was conducted to estimate the prevalence of goiter across the different regions in the country. Funnel plot asymmetry, Egger’s and Begg-Mazumdar Rank correlation tests were used to check for publication bias [26]. Two researchers independently carried out the main statistical analysis and results were crosschecked for consistency.

The risk of bias of included studies was assessed using the 10-item rating scale developed by Hoy et al. for prevalence studies [27]. Sampling, data collection, reliability and validity of study tools, case definition, and prevalence periods of the studies were assessed. Researchers categorized each study as having low risk of bias (“yes” answers to domain questions) or high risk of bias (“no” answers to domain questions). Each study was assigned a score of 1 (Yes) or 0 (No) for each domain, and these domain scores were summed to provide an overall study quality score. Scores of 8–10 were considered as having a “low risk of bias”, 6–7 a “moderate risk”, and 0–5 a “high risk” (Table 1). For the final risk of bias classification, discrepancies between the reviewers were resolved via consensus.

**Table 1:** Risk of bias assessment of eligible articles using the hoy 2012 tool

NO	Study ID	Representation	Sampling	Random selection	Non response bias	Data collection	Case Definition	Reliability and validity of study tool	Method of data collection	Prevalence period	Numerator and denominator	Summary Assessment
1	Cherinet et al.	High risk	Low risk	High risk	Low risk	Low risk	High risk	High risk	High risk	low risk	Low risk	High risk
2	Cherinet et al.	High risk	Low risk	High risk	Low risk	Low risk	High risk	High risk	High risk	low risk	Low risk	High risk
3	Berhanu et al.	Low risk	Low risk	Low risk	Low risk	Low risk	High risk	Low risk	Low risk	Low risk	Low risk	Low risk
4	Girma et al.	Low risk	Low risk	Low risk	Low risk	Low risk	High risk	Low risk	Low risk	Low risk	Low risk	Low risk
5	Mezgebu et al.	Low risk	Low risk	High risk	Low risk	High risk	High risk	High risk	Low risk	Low risk	Low risk	High risk
6	Wolka E et al.	High risk	Low risk	Low risk	Low risk	Low risk	Low risk	Low risk	Low risk	Low risk	Low risk	Low risk
7	Gebriel et al.	High risk	Low risk	High risk	Low risk	High risk	Low risk	High risk	Low risk	Low risk	Low risk	Medium risk
8	Mesele et al.	High risk	Low risk	Low risk	Low risk	Low risk	Low risk	Low risk	Low risk	Low risk	Low risk	Low risk
9	Aweke KA et al.	High risk	Low risk	Low risk	Low risk	Low risk	Low risk	Low risk	Low risk	Low risk	Low risk	Low risk
10	Kibatu et al.	High risk	High risk	Low risk	Low risk	Low risk	Low risk	Low risk	Low risk	Low risk	Low risk	Low risk
11	Enyew et al.	Low risk	Low risk	Low risk	Low risk	Low risk	Low risk	Low risk	Low risk	Low risk	Low risk	Low risk
12	Hailu et al.	High risk	Low risk	Low risk	Low risk	Low risk	High risk	Low risk	Low risk	Low risk	Low risk	Low risk
13	Sime HK et al.	High risk	Low risk	Low risk	High risk	High risk	High risk	High risk	Low risk	High risk	Low risk	High risk
14	Solomon E.	High risk	Low risk	Low risk	High risk	Low risk	Low risk	Low risk	Low risk	Low risk	Low risk	Low risk
15	Muleta et al.	Low risk	Low risk	Low risk	Low risk	Low risk	Low risk	Low risk	Low risk	Low risk	Low risk	Low risk
16	Hibstu DT et al.	Low risk	Low risk	Low risk	Low risk	Low risk	High risk	Low risk	Low risk	Low risk	Low risk	Low risk
17	Tigabu E et al.	Low risk	Low risk	Low risk	Low risk	Low risk	Low risk	Low risk	Low risk	Low risk	Low risk	Low risk
18	Ahmed A et al.	Low risk	Low risk	Low risk	Low risk	Low risk	Low risk	Low risk	Low risk	Low risk	Low risk	Low risk
19	Abebe et al.	High risk	Low risk	Low risk	High risk	Low risk	High risk	Low risk	Low risk	Low risk	Low risk	Medium risk

Risk of bias assessment tool: Yes (low risk); No (high risk)

1. Representation: Was the study population a close representation of the national population?

2. Sampling: Was the sampling frame a true or close representation of the target population?

3. Random selection: Was some form of random selection used to select the sample OR was a census undertaken?

4. Non-response bias: Was the likelihood of non-response bias minimal?

5. Data collection: Were data collected directly from the subjects?

6. Case definition: Was an acceptable case definition used in the study?

7. Reliability and validity of study tool: Was the study instrument that measured the parameter of interest show to have reliability and validity?

8. Data collection: Was the same mode of data collection used for all subjects?

9. Prevalence period: Was the length of the prevalence period for the parameter of interest appropriate?

10. Numerators and denominators: Were the numerator(s) and denominator(s) for the parameter of interest appropriate?

The overall risk of bias scored based on the number of high risk of bias per study: low risk (≤2), moderate risk (3–4), and high risk (≥5)

## Results

### Identification and description of studies

**Fig. 1:**
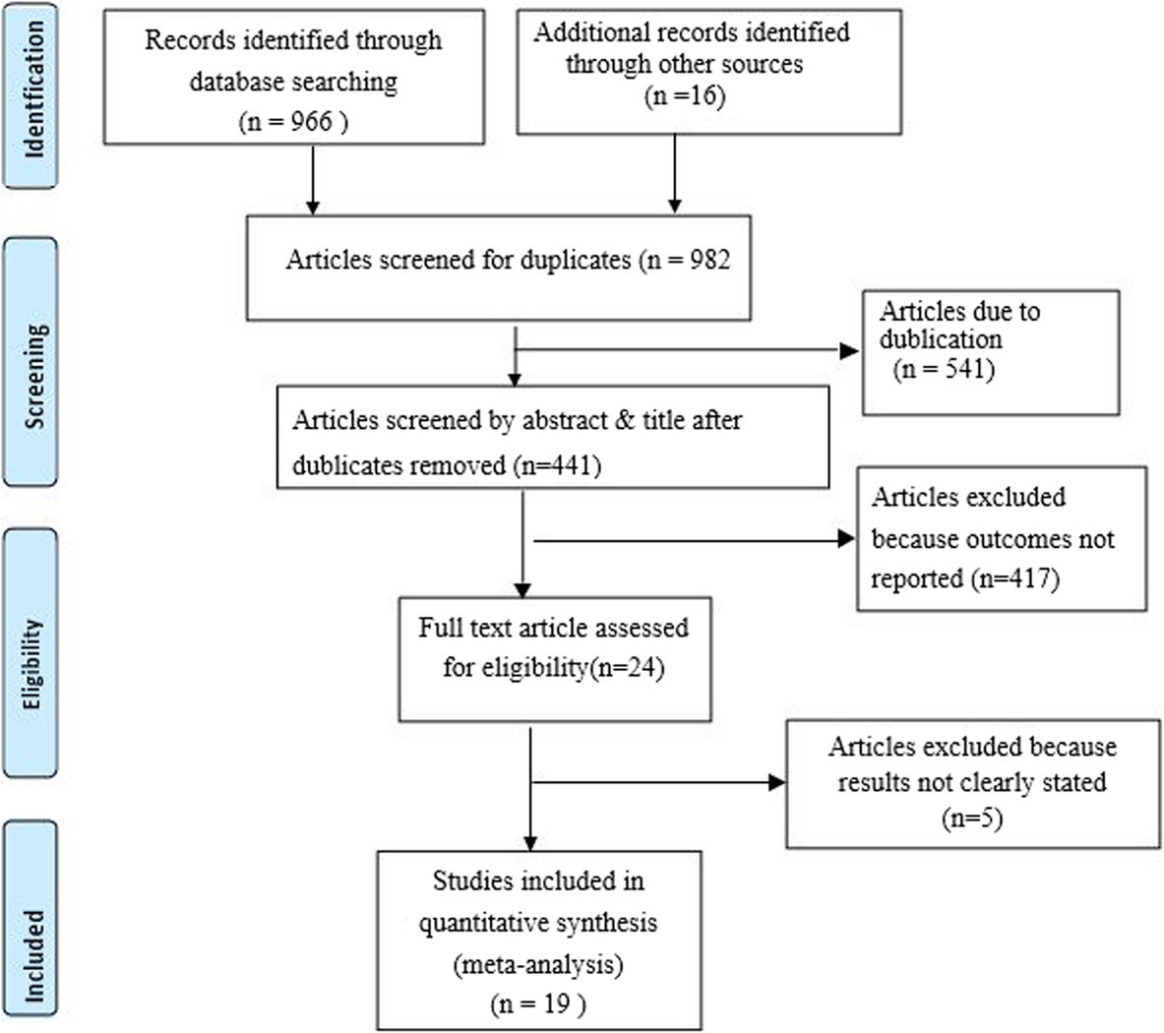
PRISMA Flow diagram showing the procedure for selecting studies for meta-analysis, 2000–2017, Ethiopia

The database search and desk review yielded a total of 982 articles. Of these, 966 articles were retrieved from PubMed, Hinari, Google Scholar, and other electronic databases. The remaining 16 were found from institutional repositories. Our search was conducted from September 1, 2018 to November 10, 2018. After reviewing the titles and abstracts, we excluded 541 articles due to duplication. In screening, we excluded 417 articles because their outcomes were not reported. The full text of the remaining 24 articles were downloaded and assessed for eligibility and quality. An additional five articles were excluded because their outcomes were not clearly stated [28–32]. The remaining 19 studies were included in the analysis (see Fig. 1).

### Characteristics of included studies

Nineteen studies with a total sample of 10,253 children (aged 6–18 years) were included in this systematic review and meta-analysis. All studies used cross-sectional study design to estimate goiter prevalence. Seven (36.8%) of the studies included in this review were from the Oromia region [33–39], Six (31.6%) were from Amhara region [40–45], four (21%) were from Southern Nations Nationalities and People’s Region (SNNPR) [34, 46–48], and the remaining two (10.5%) were from Benishangul Gumez region [49, 50]. With the exception of one study [35], all of the studies were reported in peer-reviewed journals. All of the studies used palpation as the assessment method to diagnose goiter using the WHO/UNICEF/ICIDD criteria. All of the studies reported high response rates (> 90%). The quality score of included studies ranged from 6 to 7 with a mean score of 6.67 (SD = 0.42). All of the study participants were in the age range of 6–18 years (Table 2). For risk of bias assessment, 13 of the 19 studies (68.4%) received high quality scores (≥8 points), two (10.5%) received medium scores and four (21.1%) received low scores. Regarding type of bias, 11 studies [34, 35, 37, 39, 40, 42–44, 48, 49] had high risk of representation bias; nine studies [33, 34, 37–40, 46, 47] had high risk of case definition bias; four studies [35, 39, 40] had high risk of random selection bias, and three studies [35, 39, 40] had high risk of non-response bias (Table 1). The pooled prevalence of goiter was not significantly changed after exclusion of studies with a high risk of bias.

**Table 2:** Characteristics of included studies for meta-analysis, 2000–2017, Ethiopia

Authors name	Publication Year	Source Type	Region	Outcome definition criteria	Study design	Age of study participants (in years)	Response rate (%)	Sample size	Total n outcome	Prevalence (%)	Quality Score
Cherinet A. et al. [34]	2000	Journal	Oromia	WHO/UNICEF/ICIDD	Cross-sectional	6–12	100	1825	664	36.4	6.5
Cherinet A. et al. [34]	2000	Journal	SNNPR	WHO/UNICEF/ICIDD	Cross-sectional	6–12	100	660	515	78.03	6.5
Berhanu N. et al. [33]	2004	Journal	Oromia	WHO/UNICEF/ICCIDD	Cross-sectional	6–15	100	1044	286	27.4	7
Girma M. et al. [46]	2012	Journal	SNNPR	WHO/ICCIDD/UNICEF	Cross-sectional	7–9	94.80	110	15	13.6	6
Mezgebu Y. et al. [38]	2012	Journal	Oromia	WHO/UNICEF/ICCIDD	Cross-sectional	6–12	100	389	230	59.1	7
Wolka E. et al. [48]	2014	Journal	SNNPR	WHO/UNICEF/ICCIDD	Cross-sectional	6–12	100	534	270	50.6	7
Gebriel T. et al. [49]	2014	Journal	Benishangul Gumez	WHO/UNICEF/ICCIDD	Cross-sectional	6–12	100	395	104	26.3	7
Mesele M. et al. [43]	2014	Journal	Amhara	WHO/ICCIDD/UNICEF	Cross-sectional	6–12	99.42	694	261	37.6	6
Aweke KA. et al. [42]	2014	Journal	Amhara	WHO/UNICEF/ICCIDD	Cross-sectional	6–12	100	513	281	54.7	7
Kibatu G. et al. [44]	2014	Journal	Amhara	WHO/UNICEF/ICCIDD	Cross-sectional	6–18	100	200	79	39.5	7
Enyew HD. et al. [36]	2015	Journal	Oromia	WHO/UNICEF/ICCIDD	Cross-sectional	6–12	98.20	397	200	50.6	6
Hailu S. et al. [37]	2015	Journal	Oromia	WHO criteria	Cross-sectional	6–12	93.10	393	171	43.5	6.5
Sime HK. et al. [39]	2015	Journal	Oromia	WHO/UNICEF/ICCIDD	Cross-sectional	6–12	94	709	264	37.2	7
Emiru S. [35]	2016	Institutional repository	Oromia	WHO/UNICEF/ICCIDD	Cross-sectional	7–12	100	270	63	23.3	6
Muleta F. et al. [50]	2016	Journal	Benishangul Gumez	WHO/UNICEF/ICCIDD	Cross-sectional	6–18	100	200	79	39.5	7
Hibstu DT. et al. [47]	2017	Journal	SNNPR	WHO	Cross-sectional	6–12	99.40	358	126	35.2	7
Tigabu E. et al. [45]	2017	Journal	Amhara	WHO/UNICEF/ICCIDD	Cross-sectional	7–12	97.60	443	275	62.1	7
Ahmed A. et al. [41]	2017	Journal	Amhara	WHO/UNICEF/ICCIDD	Cross-sectional	6–15	100	384	113	29.1	7
Abebe Z. et al. [40]	2017	Journal	Amhara	WHO	Cross-sectional	6–12	100	735	214	29.1	7

### Publication bias

**Fig. 2:**
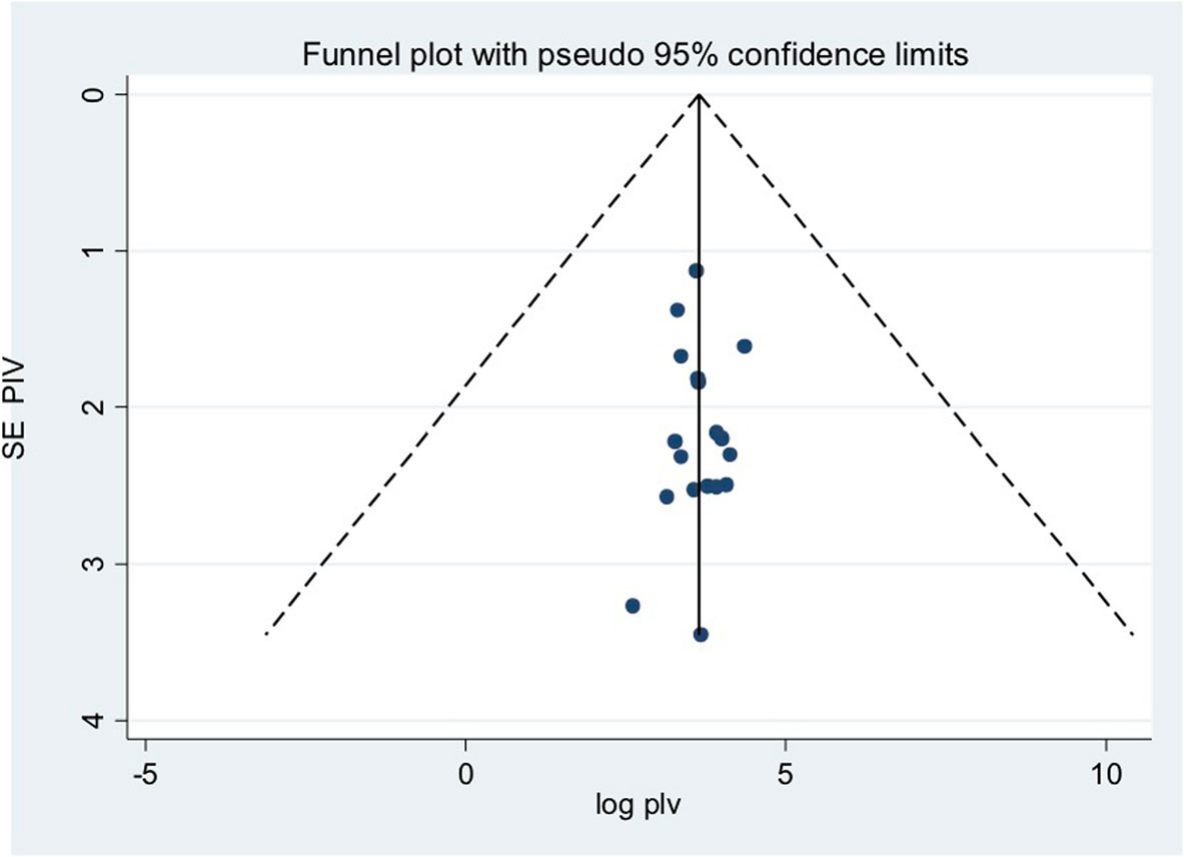
Meta funnel presentation of the prevalence of goiter among children, 2000–2017, Ethiopia

Both funnel plots of precision asymmetry and the Egger’s test of the intercept indicated the absence of publication bias in the included studies. Visual examination of the funnel plot showed a symmetric distribution of studies (Fig. 2). Additionally, Egger’s test of the intercept was − 0.004 (95% CI: − 0.3–0.3) *p* > 0.05, suggesting that publication bias estimates were not statistically significant.

### Prevalence of goiter among children in Ethiopia

**Fig. 3:**
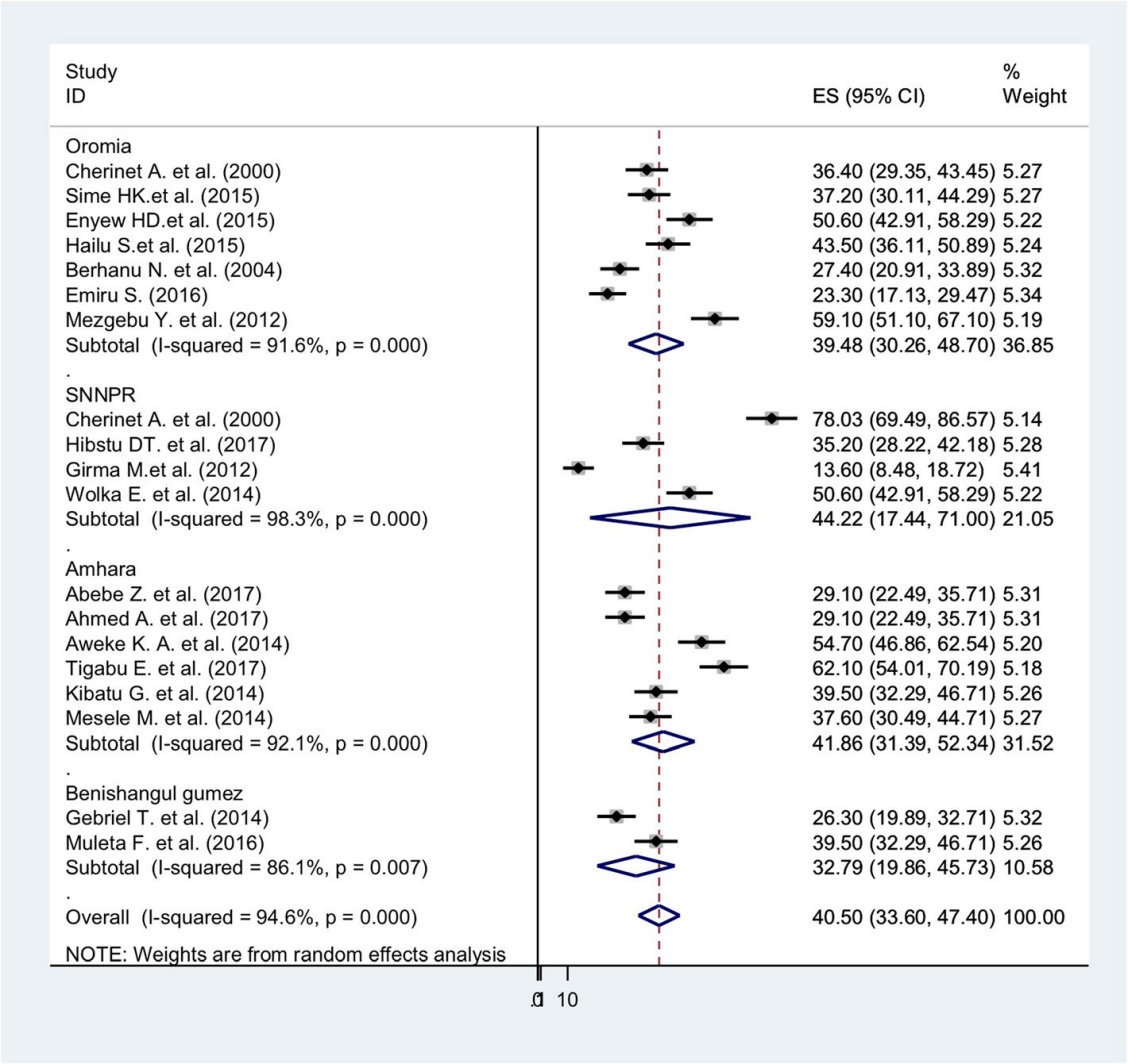
Forest plot showing the pooled prevalence of goiter in regions among male children in Ethiopia, 2000 to 2017

Overall, the pooled prevalence of goiter among children in Ethiopia was 40.50% (95% CI: 33.6–47.40) (Fig. 3). The lowest (13.6%) [46] and highest (78.03%) [34] prevalence reported of were found in studies conducted in SNNPR. Because the I^2^ static test for heterogeneity indicated that the studies differed significantly (I^2^ = 94.6%, *p* < 0.05) and because theoretically we expected large differences in the study settings and socio-economic contexts, we fitted a DerSimonian and Laird random effect model to estimate the pooled prevalence of goiter [51, 52]. The studies with the largest weight were Girma et al.’s [46], Emiru et al.’s [35], and Berhanu et al.’s [33] with respective weights of 5.41, 5.34, and 5.32%. A slightly smaller weight of 5.14% was given to Cherinet et al.’s study [38].

In the sub-group analysis by region, the highest pooled prevalence of goiter was found in SNNPR, 44.22% (95% CI: 17.44, 71), and in Amhara region, 41.86% (95% CI: 31.39–52.34). The pooled prevalence of goiter among children in Oromia region was 39.48% (95% CI: 30.26–48.70), and in Benishangul Gumez, 32.79% (95% CI: 19.86–45.73) (Fig. 3).

The sub-group analysis indicated the presence of heterogeneity across the studies. To identify the source of heterogeneity, we conducted meta-regression and sensitivity analysis. The meta-regression analysis was conducted using the following study covariates: publication years, sample size, and region. However, the results showed that none of these variables were a statically significant source of heterogeneity.

We also performed a sensitivity analyses to examine the influence of each study on the overall effect size. No single study significantly affected the overall pooled estimate of goiter.

### Sex difference in goiter prevalence

The average pooled estimate of goiter among female and male children was 44.34% (95% CI: 35.02–53.66) and 32.88% (95% CI: 25.32–40.45), respectively. However, the difference in prevalence by sex was not statistically significant, as the 95% confidence intervals overlap. Studies in SNNPR reported the highest pooled prevalence of goiter among both female and male children: 55.09% (95% CI: 13.57–92.62) and 42.07% (95% CI: 3.70–80.44) respectively. Studies in Benishangul Gumez had the lowest prevalence for both sexes: 23.57% (95% CI: 19.20–27.95) for female children and 22.21% (95% CI: 13.69–30.73) for male children (Table 3).

**Table 3:** Distribution of goiter based on sex and region in studies conducted from 2000 to 2017, in Ethiopia

Regions	Male	Female
Amhara	31.7% (95% CI: 20.0–43.3)	47% (95% CI: 30.1–63.8)
Oromia	33.2% (95% CI: 23.3–43.1)	44.4% (95% CI: 32.8–56%)
SNNPR	42.1% (95% CI: 3.7–80.4)	55.1% (95% CI: 13.6–92.6)
Benishangul Gumez	22.21% (95% CI: 13.7–30.7)	23.6% (95% CI: 19.2–28.0)

### Factors associated with goiter among children in Ethiopia

**Fig. 4:**
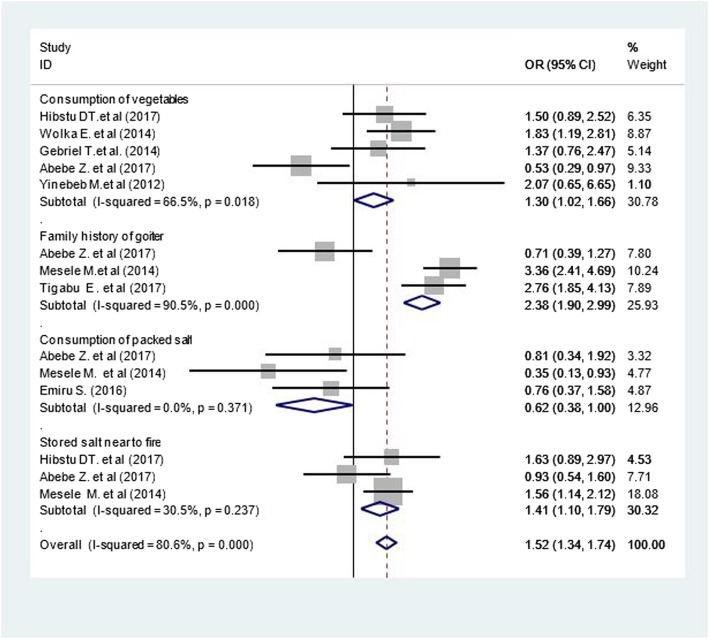
Forest plot showing factors associated with goiter among children in Ethiopia, 2000 to 2017

Five studies [38, 40, 47–49] were assessed for the association between high vegetable consumption and goiter. Children who consume vegetables three or more times per week were significantly more likely to develop goiter than those who consumed less, OR = 1.3 (95% CI: 1.02–1.66) (Fig. 4).

Our analysis of three studies [40, 43, 45] found that children who have family history of goiter had more than double the odds of having goiter, OR = 2.38 (95% CI: 1.90–2.99). Our examination of three studies [35, 40, 43] found no association between the consumption of packaged salt and goiter in children OR = 0.62 (95% CI: 0.38–1.00). However, the three studies examining salt storage [35, 43, 47] found that storing salt next to fires had a modest but significant positive association with goiter in children OR = 1.41 (95% CI: 1.10–1.79).

Heterogeneity tests showed evidence of high and moderate heterogeneity in studies that assessed vegetable consumption and family history as factors respectively (I^2^ of 90.55 and 66.5% with *p*-value< 0.05). The publication bias was also significant for all factors (Begg’s test = 0.033 and Egger’s test = 0.015).

## Discussion

This meta-analysis and systematic review was conducted to estimate the pooled prevalence of goiter among children in Ethiopia. We also assessed factors associated with goiter among children using the available published and unpublished studies.

We found a high prevalence of goiter among children, with an overall pooled prevalence of 40.5% (95% CI: 33.6–47.41). This high prevalence meets WHO’s threshold for endemicity [3] and implies the presence of moderate to severe iodine deficiency in the country [53]. Our estimated prevalence is similar to the 39.9% rate (95% CI: 38.6–41.20) found in a nationwide, community-based, cross-sectional study conducted in 2005 [11] and a systematic review of the literature on progress in eliminating iodine deficiency in Ethiopia which found a 35% child prevalence of goiter [20]. Additionally, a survey among school children carried out in 2016 found that 48% of school-age children had iodine deficiency [54]. Assuming that these estimates and ours are accurate, it is striking that the prevalence of goiter has not significantly decreased over time in line with improvements in the distribution of iodized salt in the country.

Low consumption of iodized salt and inadequate levels of iodine in iodized salt may account for these persistently high rates of goiter. A previous Ethiopian study found that one-third of salt samples in the study area were not adequately iodized [55] and another that a significant proportion (22.2 to 64.6%) of the households in Ethiopia used inadequately iodized salt; well below the WHO recommended level [56]. In other low- and middle-income countries with more robust salt iodization programs we see dramatically lower prevalence of goiter. For example, in Lesotho where 94.4% of salt samples are iodized, the goiter prevalence in children has been estimated to be 4.9% [57]. Similarly, in India, a large population-based study found 80% adequate iodized salt coverage [58] and community-based studies have found goiter prevalence in children ranging from 4.29–13.80% [58, 59].

Ethiopia’s high prevalence of goiter among children is a significant public health problem. The presence of goiter with iodine deficiency (including mild forms of deficiency) is one of the main causes of delayed mental development among children [58]. IDDs such as goiter create significant short- and long-term physical, mental and socio-economic harm and their continued presence in Ethiopia underscores the need to reinvigorate the country’s universal salt iodization program [60]. It is well established that continuous iodized salt supplementation can contribute significantly to the prevention of goiter and cognitive impairment in children [53, 61, 62].

Providing consistent supplementation will require more training and better equipment for the many small-scale local salt producers in order to improve adequate idolization of salt [63]. As important as supporting small-scale salt producers will be increasing investment in large, “industrial-scale iodization factories” [63]. It must also be noted that even in settings with well-functioning universal salt iodization programs infants may not consume enough salt to prevent IDDs, therefore, additional supplementation for pregnant women and infants may be necessary [10].

We found large regional variation in the prevalence of goiter among children. Regional differences in prevalence may be due to regional differences in soil composition; differences in the consumption of cruciferous vegetables, cassava leaves and wild edible plants that may affect thyroid function; and in differences in the level of bacterial contamination of drinking water. For example, SNNPR, were we saw the highest goiter prevalence (44.22%), has high consumption of wild edible plants and cassava leaves [64, 65] and many of the SNNPR studies analyzed in this review were conducted in areas where drinking water has significant bacterial contamination [34], which may significantly increase the risk of goiter [66]. The second highest prevalence of goiter was reported from Amhara region (41.9% prevalence) which is a mostly highland area with nutrient poor, eroded soils [67]. In contrast, the Oromia (39.5% prevalence) and Benishangul-Gumuz regions (32.79% prevalence) are lowland forest and farming areas that should have more iodine in the soil [68, 69]. The observed regional variation in prevalence suggests that when rolling out improved national iodization programs, SNNPR and Amhara may deserve special attention.

The prevalence of goiter among females was roughly 11 percentage points larger than that reported among males. This finding is unsurprising as it is well established in Ethiopian [20] and global studies [14] that women have higher rates of goiter than men and that this difference is most pronounced in iodine deficient areas and among younger age groups, particularly among those in late puberty [5, 6]. These sex differences in goiter prevalence may be due to differences between the sexes in levels of the hormones and sex steroids that affect thyroid function. These differences may be particularly pronounced during puberty and menopause [5, 70]. Given young women’s relatively higher risk, stand-alone, school- and community-based health education and supplementation programs that target this population may deserve consideration.

Our study also found that the consumption of vegetables and storing salt near to fires modestly increased the odds of goiter among children, and that a family history of goiter had a large estimated positive impact on the odds of having goiter. The consumption of packaged vs. loose salt had no significant association with goiter among children.

The findings on vegetable consumption are somewhat expected as it has long been posited that consumption of goitrogenic foods such as cabbage and cassava increase concentrations of thiocyanate, which might interfere with iodine transport [71]. Although recent epidemiological and experimental studies provide little evidence for this relationship (and may indeed suggest that thiocyanate improves thyroid function) [72, 73], animal studies offer some support for the theory that high consumption of goitrogenic foods might lead to hypothyroidism. In animal studies, feeding cassava that metabolized to thiocyanate in the absence of adequate iodine supplementation causes hypo functioning of the thyroid gland [74]. In recent physiological studies the morphological changes after prolonged consumption of goitrogenic foods were characterized by replacement of colloid containing follicles with cuboidal cells with distinct nucleus that showed hypertrophy and hyperplasia. These morphological changes were also accompanied by inhibition of thyroid peroxidase and 5′ monodeiodinase with simultaneous decrease in serum thyroxine (T4), and triiodothyronine (T3) levels. These anatomical and physiological changes may lead to absolute biochemical hypothyroidism [73–76].

Larger more robust studies of the risks associated with diets that are extremely high in goitrogenic vegetables and assessments of how these risks are balanced with benefits may be warranted. Such studies would require rigorous research designs to control for possible confounding between very high consumption of vegetables such as cassava, which are staples of the poor during times of food insecurity, and factors such as poverty that might also be associated with low-iodine consumption and other micro-nutrient deficiencies that exacerbate IDDs.

We found that the prevalence of goiter was more pronounced among families who stored salt near fires, OR = 1.41, but this estimate was not very precise as our confidence interval ranged from 1.1 (almost no effect on the odds of goiter) to 1.79 (almost double the effect). Other studies have suggested that iodine concentration can be significantly lower when salt was stored near a fireplace before use [77] due to chemical changes that occur with rapid evaporation [19, 78]. The consumption of packaged salt had no significant association with goiter prevalence. This finding was somewhat surprising, as, intuitively one would assume that packaged salt would have higher iodine content. However, other factors such as duration of salt storage, and whether salt is covered in storage might also affect iodine content and reduce the importance of packaging. In short, the importance of home storage and packaging practices in preventing goiter is unclear. As a precaution, it may be useful to include instruction on salt storage in nutrition health education until the effects of storage on iodine deficiency outcomes are better established.

The prevalence of goiter was significantly larger among children who had a family history of goiter, OR = 2.38 (95% CI: 1.90–3.99). This finding is expected as family clustering of goiter has been common historically [79]. A complex, “multifactorial” interaction of genetic and shared environmental factors are thought to a explain higher rates of goiter among people with a family history [79].

In our study, a risk of bias assessment showed that 13 (68.4%) studies had high quality scores and two (10.5%) had moderate quality scores. Representation and case-definition biases were the most commonly noted. To determine the influence of low methodological quality/high risk of bias on our estimates we estimated pooled prevalence without the low-quality studies. The confidence intervals of our estimates of pooled prevalence with and without these studies overlapped, indicating no significant difference between them. These results suggest that the majority of the primary study authors have met quality standards and lend credibility to our findings.

## Limitations

Due to the absence of data, crude odds ratios were used to estimate factors related to goiter, which prevented us from excluding confounding factors. Our discussion of factors associated with goiter would be best interpreted keeping this important limitation in mind. In addition, although we screened all of the studies in this review for quality, we must note that most were published in very small journals where it is difficult to gauge the quality of the peer review process, and most had only modest quality scores.

## Conclusion

This meta-analysis found that the prevalence of goiter among children in Ethiopia is high, and that goiter may be endemic in the country according to WHO criteria. Goiter among children was significantly associated with consuming vegetables three or more times per week, the presence of family history of goiter, and storing salt near a fire. Our results suggest that more attention should be given to strengthening the government’s national salt iodization program. Future research should investigate household-level factors that contribute to high goiter prevalence even when there is high coverage of adequately iodized salt. In particular, additional research on appropriate salt storage and the risks of consuming goitrogenic foods require more robust investigation.

## Data Availability

All data are available in the manuscript.

## Appendix

### PubMed Advanced Search Terms

(((((("epidemiology"[Subheading] OR "epidemiology"[All Fields] OR "epidemiology"[MeSH Terms]) OR ("epidemiology"[Subheading] OR "epidemiology"[All Fields] OR "prevalence"[All Fields] OR "prevalence"[MeSH Terms])) AND ("goitre"[All Fields] OR "goiter"[MeSH Terms] OR "goiter"[All Fields])) AND (associated[All Fields] AND factors[All Fields])) OR (determinant[All Fields] AND factors[All Fields])) AND ("child"[MeSH Terms] OR "child"[All Fields])) OR ("child"[MeSH Terms] OR "child"[All Fields] OR "children"[All Fields])) AND ("ethiopia"[MeSH Terms] OR "ethiopia"[All Fields])

## Abbreviations

CI: Confidence interval
ICCIDD: International Council for Control of Iodine Deficiency Disorders
IDD: Iodine Deficiency Disorders
NOS: Newcastle-Ottawa Scale
OR: Odds ratio
SNNPR: Southern Nations, Nationalities, and Peoples’ Region
UNICEF: United Nations International Children’s Emergency Fund
WHO: World Health Organization
